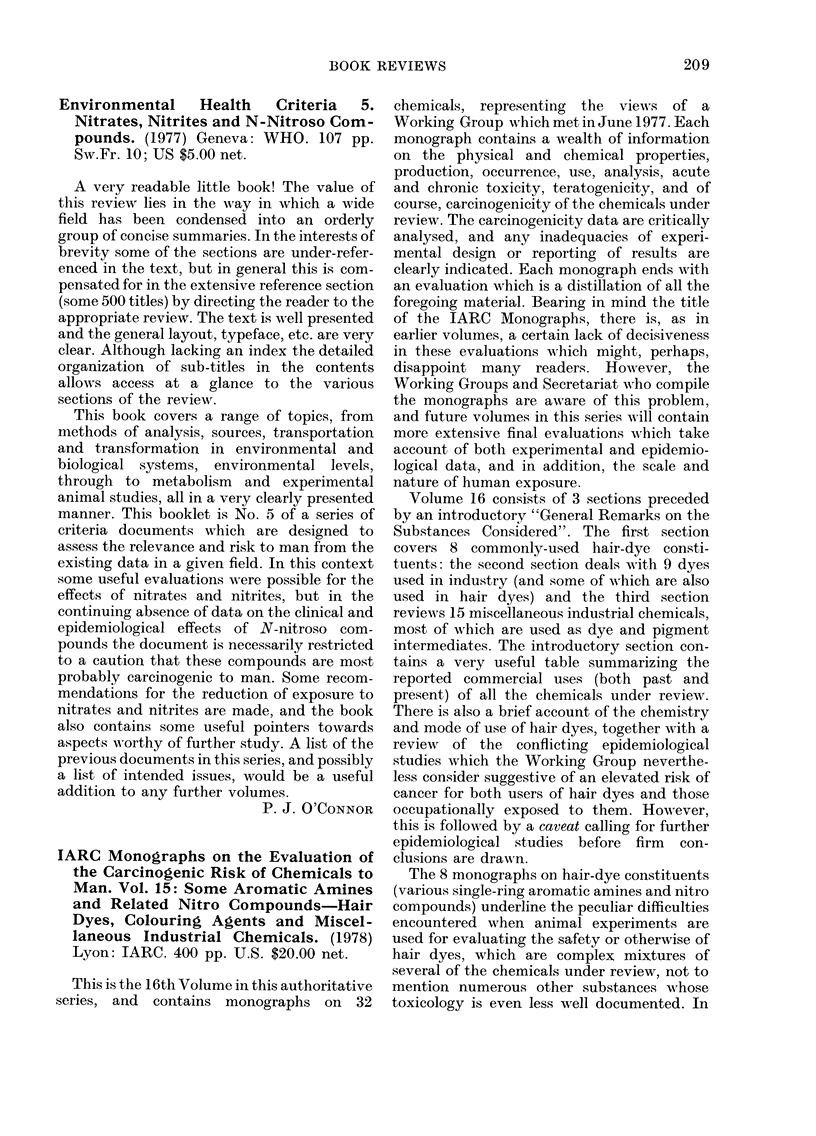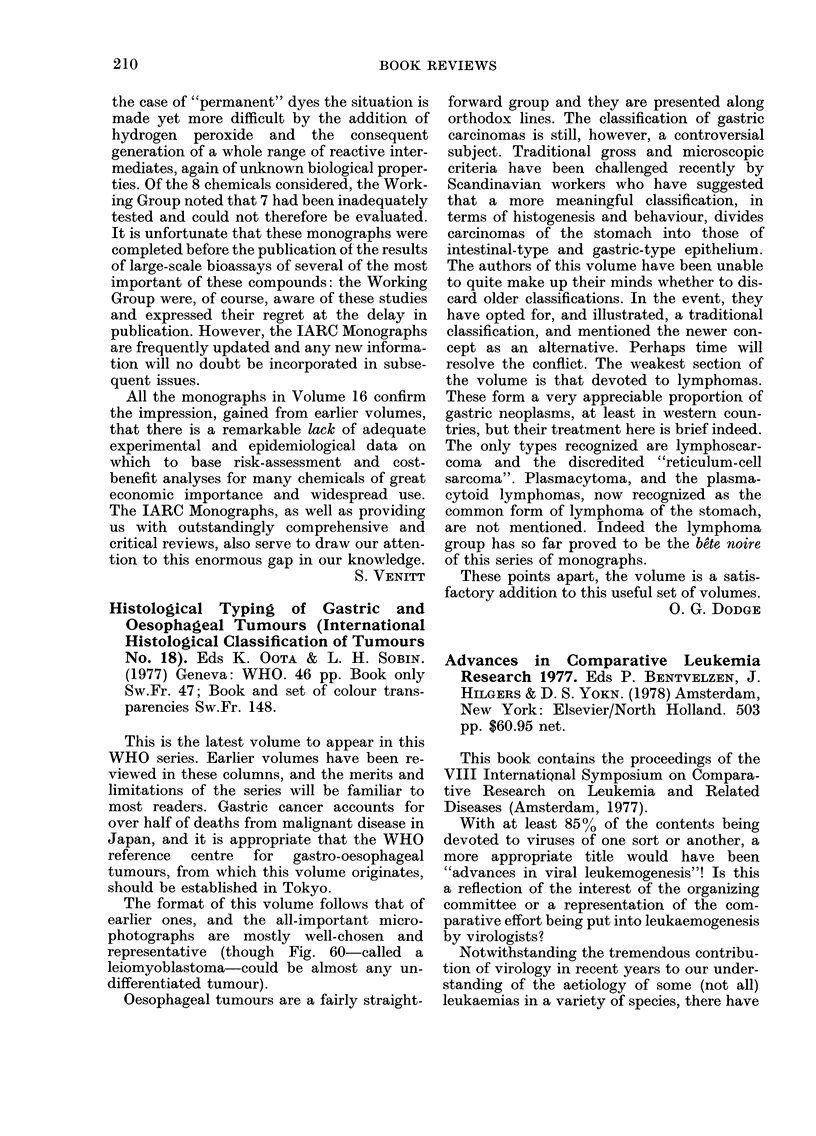# IARC Monographs on the Evaluation of the Carcinogenic Risk of Chemicals to Man. Vol. 15: Some Aromatic Amines and Related Nitro Compounds—Hair Dyes, Colouring Agents and Miscellaneous Industrial Chemicals

**Published:** 1979-02

**Authors:** S. Venitt


**IARC Monographs on the Evaluation of the Carcinogenic Risk of Chemicals to Man. Vol. 15: Some Aromatic Amines and Related Nitro Compounds—Hair Dyes, Colouring Agents and Miscellaneous Industrial Chemicals.** (1978) Lyon: IARC. 400 pp. U.S. $20.00 net.

This is the 16th Volume in this authoritative series, and contains monographs on 32 chemicals, representing the views of a Working Group which met in June 1977. Each monograph contains a wealth of information on the physical and chemical properties, production, occurrence, use, analysis, acute and chronic toxicity, teratogenicity, and of course, carcinogenicity of the chemicals under review. The carcinogenicity data are critically analysed, and any inadequacies of experimental design or reporting of results are clearly indicated. Each monograph ends with an evaluation which is a distillation of all the foregoing material. Bearing in mind the title of the IARC Monographs, there is, as in earlier volumes, a certain lack of decisiveness in these evaluations which might, perhaps, disappoint many readers. However, the Working Groups and Secretariat who compile the monographs are aware of this problem, and future volumes in this series will contain more extensive final evaluations which take account of both experimental and epidemiological data, and in addition, the scale and nature of human exposure.

Volume 16 consists of 3 sections preceded by an introductory "General Remarks on the Substances Considered". The first section covers 8 commonly-used hair-dye constituents: the second section deals with 9 dyes used in industry (and some of which are also used in hair dyes) and the third section reviews 15 miscellaneous industrial chemicals, most of which are used as dye and pigment intermediates. The introductory section contains a very useful table summarizing the reported commercial uses (both past and present) of all the chemicals under review. There is also a brief account of the chemistry and mode of use of hair dyes, together with a review of the conflicting epidemiological studies which the Working Group nevertheless consider suggestive of an elevated risk of cancer for both users of hair dyes and those occupationally exposed to them. However, this is followed by a *caveat* calling for further epidemiological studies before firm conclusions are drawn.

The 8 monographs on hair-dye constituents (various single-ring aromatic amines and nitro compounds) underline the peculiar difficulties encountered when animal experiments are used for evaluating the safety or otherwise of hair dyes, which are complex mixtures of several of the chemicals under review, not to mention numerous other substances whose toxicology is even less well documented. In the case of "permanent" dyes the situation is made yet more difficult by the addition of hydrogen peroxide and the consequent generation of a whole range of reactive intermediates, again of unknown biological properties. Of the 8 chemicals considered, the Working Group noted that 7 had been inadequately tested and could not therefore be evaluated. It is unfortunate that these monographs were completed before the publication of the results of large-scale bioassays of several of the most important of these compounds: the Working Group were, of course, aware of these studies and expressed their regret at the delay in publication. However, the IARC Monographs are frequently updated and any new information will no doubt be incorporated in subsequent issues.

All the monographs in Volume 16 confirm the impression, gained from earlier volumes, that there is a remarkable *lack* of adequate experimental and epidemiological data on which to base risk-assessment and cost-benefit analyses for many chemicals of great economic importance and widespread use. The IARC Monographs, as well as providing us with outstandingly comprehensive and critical reviews, also serve to draw our attention to this enormous gap in our knowledge.

S. Venitt